# Supplementary material for: Plant interactions associated with a directional shift in the richness range size relationship during the Glacial-Holocene transition in the Arctic
Source: Nat Commun. 2025 Jan 28;16:1128. doi: 10.1038/s41467-025-56176-3 (PMC11775137; doi:10.1038/s41467-025-56176-3)
Supplement: Supplementary file 1 — Supplementary Information [file 41467_2025_56176_MOESM1_ESM.pdf]

Plant interactions associated with a directional shift in the richness range size relationship during the Glacial-Holocene transition in the Arctic

Supplementary Information

Ying Liu[1, 2], Simeon Lisovski[1], Jérémy Courtin[1], Kathleen R. Stoof-Leichsenring[1], Ulrike Herzschuh[1,2,3*]

[1] Alfred Wegener Institute Helmholtz Centre for Polar and Marine Research, Polar Terrestrial Environmental Systems, Telegrafenberg, 14473 Potsdam, Germany

[2]Institute of Environmental Science and Geography, University of Potsdam, 14476 Potsdam, Germany

[3]Institute of Biology and Biochemistry, University of Potsdam, 14476 Potsdam, Germany

***Correspondence**

Ulrike Herzschuh: Ulrike.Herzschuh@awi.de

Contents

Supplementary Notes:

Supplementary Note 1: Sampling site description.

Supplementary Note 2: Subsampling, DNA extraction, and amplification.

Supplementary Note 3: Area of Occupancy (AOO) and Extent of Occurrence (EOO) method comparison.

Supplementary Note 4: Time-windows and choice of grid cell size.

Supplementary Note 5: Assessing the impact of extinction on the richness-range size relationship

Supplementary Figures:

Supplementary Figure 1: Comparison of mean range-size estimates using the Area of Occupancy (AOO) and Extent of Occurrence (EOO) methods.

Supplementary Figure 2: Comparison of the change in slope of the richness to mean range-size relationship in time-windows based on the Area of Occupancy (AOO) and Extent of Occurrence (EOO) methods.

Supplementary Figure 3: Richness (a) and range-size change over 5,000-year running time-windows for the Area of Occupancy (AOO; b) and Extent of Occurrence (EOO) methods (c).

Supplementary Figure 4: Plant taxa richness to range-size relationships (with 95% confidence intervals) for time-windows ranging from 10,000-year (10 ka) to 2,000-year (2 ka), over the last 30,000 years, inferred from lake sedimentary ancient DNA collected from northeast Siberia and Alaska region.

Supplementary Figure 5: The modern plant richness to range-size relationship across space in the northeast Siberia and Alaska region, based on modern plant taxa occurrences in grid cells of four sizes from 100 km × 100 km grid cells to 500 km × 500 km grid cells (a, c, e, g). Comparison of the same taxa range-size between two calculation methods: based on the sum of occupied grid cells centred on the seven lakes, and the sum of occupied grid cells in

northeast Siberia and Alaska region, for four grid cell sizes ranging from 100 km × 100 km to 500 km × 500 km (b, d, f, h).

Supplementary Figure 6: Correlation network of plant groups.

Supplementary Figure 7: Plant taxa richness to range-size relationships (with confidence intervals) per 5,000-year time-windows over the last 30,000 years, inferred from the taxa that occur in both the glacial and Holocene periods across northeast Siberia and Alaska.

Supplementary Tables:

Supplementary Table 1. The slope, R squared, and p-value of the richness to mean range-size (Area of Occupancy method) linear regression model based on 5,000-year running time-windows.

Supplementary Table 2. The slope, R squared, and p-value of the richness to mean range-size (Extent of Occurrence method) linear regression model based on 5,000-year running time-windows.

Supplementary Notes

Supplementary Note 1: Sampling site description

Lake Billyakh (65°17′N, 126°47′E; 340 m a.s.l.) [1]is situated in the western part of the Verkhoyansk Mountains, approximately 140 km south of the Arctic Circle. Its area is ca 23 km^2, the lake water depth is 8 m, and it has a maximum depth of about 25 m. Fed by precipitation and several small creeks and streams, it experiences a harsh climate, with mean January temperatures plummeting to around −40°C, contrasting with mean July temperatures ranging from 15−19°C. Recorded annual precipitation is recorded between 300 and 400 mm. Currently, the area is dominated by cold deciduous forests and woodlands, primarily comprising Larch (*Larix*).

Lake Bolshoe Toko (56°05′ N, 130°90′ E, 903 m a.s.l.) [2] is located in a depression of tectonic and glacial origin, on the northern flank of the eastern Stanovoy Mountain Range. It is bordered by moraines of three glaciers at its northeastern margins. It is 15.4 km long and 7.4 km wide, with a maximum water depth of about 80 m and a surface area of 82.6 km^2. The mean annual air temperature in the study region is 11.2°C, ranging from −65°C in January to +34°C in July, and annual precipitation varies from 276 to 579 mm. Soil cover is thin and contains large amounts of gravel. Northern taiga dominates the study area, forests consist of *Larix*, *Picea*, and *Pinus*.

Lake E5 (68.641667N, 149.457706 W, 795 m a.s.l.) [3] is situated in the northern foothills of the Brooks Range, northern Alaska, it is located on an older glacial landscape (>125 ka BP), referred to as the Sagavanirktok, that escaped glaciation during the last Glacial maximum (LGM). It is a small lake of ~650 m long x 310 m wide at its widest point, and a surface area of 0.1 km^2. Vegetation, primarily tussock tundra, blankets the Sagavanirktok River moraine surrounding Lake E5, while a scattering of large erratic boulders punctuates the vegetated surface along the ridgetop southwest of the lake, rising 1−2 m above the terrain.

Lake Emanda (65°17.649′N, 135°45.554′E, 671 m a.s.l.) [4] is a heart-shaped lake in the Yana Highlands. It spans 7.5 km in length and 6.5 km in width, covering a surface area of 33.1 km^2,

with a maximum water depth of 15 m. The climate is characterized as continental subarctic, marked by significant seasonal temperature variations and an average annual precipitation of 233 mm. Winters are dominated by the Siberian High-Pressure system, resulting in mean January temperatures of −47.2°C and thin snow cover. Summers are influenced by the Asiatic Thermal Low-Pressure system and a North Pacific High-Pressure system, leading to short but relatively warm periods with mean July temperatures of 13.1 °C and increased precipitation, reaching 40–50 mm per month from June to August. The soil surrounding the lake is characterized by permafrost. Vegetation primarily consists of sparse mountain larch (*Larix cajanderi*) forests, with lichen (*Flavocetraria cucullata*, *Cladina arbuscula*), *Betula exilis*, and *Pinus pumila*, as well as *Vaccinium*, moss, and lichen communities (*V. vitis-idaea*, *V. uliginosum*, *Aulacomnium turgidum*, *Sphagnum* spp., *Flavocetraria cucullata*).

Lake Ilirney (67°21′N, 168°19′E, 1790 m a.s.l.) [5] is situated in Chukotka, Russia's far east, bounded by the Anadyr Mountains to the north. The basin spans 12 km in length and 3.6 km at its widest point, following a northeast to southwest orientation along a major structural feature. It comprises two sub-basins: a larger depocenter in the southwest and a smaller basin in the northeast, separated by a north–south oriented bathymetric high. Maximum water depths reach 44 m in the southwest basin and 25 m in the northeast basin. The catchment area covers 1214 km^2. Ilirney experiences a strongly continental climate, with mean annual temperature of −13.5 °C, and January and July averages of −33.4 °C and 12.1 °C, respectively. Regional snowfall amounts to 110 mm of water, while June–September precipitation totals 70 mm. The lake zone is covered by continuous permafrost and located at the tundra-taiga ecotone boundary, with diverse vegetation in its vicinity.

Lake Rauchuagytgyn (67°49′N, 168°44′E, 619 m a.s.l.) [6] lies within the U-shaped Rauchua mountain valley, eroded by glacial activity in the northwestern Anadyr Mountains of Chukotka. Stretching approximately 4.35 km in length, with a maximum depth of ~36 m, it covers an area of 6.24 km^2, draining a catchment area of about 215 km^2. The region experiences a mean annual air temperature of around −11.8 °C, with mean July temperatures reaching 13 °C and mean January temperatures dropping to −30 °C. The short growing season spans

approximately 100 days per year, with annual precipitation averaging ~200 mm. Located within the continuous permafrost zone, evidence of permafrost processes is from thermokarst lakes and ice-wedge polygons in the inflow regions. The lake vicinity is characterized by prostrate dwarf shrubs, and herbaceous and graminoid tundra, primarily *Dryas octopetala*, along with various taxa from Poaceae, Fabaceae, and Asteraceae families. This transitions to barren land at higher elevations, while forest tundra dominates at lower elevations along river valleys and favourable habitats.

Lake Levinson-Lessing (74°27′54″N, 98°39′58″E, 47 m a.s.l.) [7] lies in the southern region of the Byrranga Mountains, which formed during the Hercynian Orogeny and has undergone numerous tectonic modifications. The lake spans 15 km in length and up to 2 km in width, covering an area of ~25 km², with a catchment of ~515 km² and a maximum water depth of ~120 m. The climate is characterized by prolonged winters with temperatures dropping to ~−34°C in January and brief summers with temperatures reaching ~6 °C in July, accompanied by an annual precipitation of ~250 mm. Situated within the continuous permafrost zone, the lake basin straddles the border between the subarctic and Arctic tundra zones, predominantly inhabited by grass, sedge, and herb communities.

The age models for all the sediment records follow previous studies [1-7].

Supplementary Note 2: Subsampling, DNA extraction, and amplification

The subsampling of lake sediment cores for sedaDNA analysis was conducted in a clean climate chamber of the Helmholtz Centre Potsdam-German Research Centre for Geosciences (GFZ) with a room temperature of 4°C. The risk of contamination with modern DNA was reduced by covering up personal clothing and using sterile subsampling equipment. The surface layer of the halved core was scraped with a sterile scalpel. DNA samples were taken from the untouched inner part of the cores and stored at −20°C.

DNA extractions were performed in the dedicated paleogenetic laboratory at the Alfred Wegener Institute, Helmholtz Centre for Polar and Marine Research, Potsdam, Germany, following strict ancient DNA protocols. Each extraction batch included nine sediment samples plus one extraction control and followed the modified protocol of the DNeasy PowerSoilMax Soil Kit (Qiagen, Germany). The samples were added to a mixture of 1.2 mL of C1 buffer, 0.4 mL of 2 mg/mL Proteinase K (VWR International, Germany), and 0.5 mL of 1M dithiothreitol (VWR International, Germany) in PowerBead tubes, and vortexed for 30 seconds at maximum speed. Then, the samples were homogenized for 50 seconds using the MP Biomedicals™ FastPrep™-24 Bead-Beating device at maximum speed and incubated at 56°C in a rocking shaker overnight. Subsequent steps followed the instructions of the manufacturer Qiagen. Final DNA extracts were eluted in varied between 1.6 and 2.0 mL C6 buffer, where 1 mL (except for samples from Lake Bolshoe Toko) was concentrated using the GeneJET PCR Purification Kit (Thermo Fisher Scientific) with 100 µl to 50 µL elution buffer. The purified DNA concentration was measured using the ds-DNA BR Assay Kits and the Qubit® 2.0 fluorometer (Invitrogen, USA). Then, the DNA solution was diluted to 3 ng/µL for the subsequent PCR process.

PCR reactions were performed using the g (GGGCAATCCTGAGCCAA) and h (CCATTGAGTCTCTGCACCTATC) primers targeting the vascular plant *trn*L p6-loop locus of the chloroplast genome[8]. Both primers were tagged with an N-8bp tag at the 5’ end, which allowed indexing of the PCR samples and subsequent demultiplexing. PCR reactions were conducted in a final volume of 25 µL, using 3 µL of DNA extract diluted to 3 ng/µL as a template, and 12.8 µL H_2O, 2.5 µL Taq DNA Polymerase PCR Puffer (10x), 0.25 mM dNTPS, 0.8 mg Bovine Serum Albumin, 2 mM $MgSO_4$ (Invitrogen, USA), 1 U Platinum® Taq High Fidelity DNA Polymerase (Invitrogen, USA), and 0.2 mM of each primer. PCRs were run with initial denaturation at 94°C for 5 min, followed by 40 cycles of 94°C for 30 s, 50°C (annealing temperature) for 30 s, 68°C (elongation temperature) for 30 s, and final extension at 72°C for 10 min. An extraction control (blank) and one no-template control (NTC) were included in each

PCR batch to identify possible contamination during extraction and PCR set-up. Finally, three positive PCR replicates for each sediment sample were aggregated and purified using the MinElute PCR Purification Kit (Qiagen, Germany), following the recommended protocol, and eluted in 20 µL of elution buffer. The DNA concentrations were quantified with the ds-DNA BR Assay Kits and the Qubit® 2.0 fluorometer (Invitrogen, USA). To avoid bias based on differences in DNA concentration between samples, all replicates were pooled in equimolar concentrations for each core. For each core, all extraction blanks and NTCs were included in the sequencing run, even though they were negative in the PCRs. Each sequencing run was performed by Fasteris SA sequencing service (Switzerland) with the paired-end sequencing on a HiSeq or NextSeq Illumina platform.

Supplementary Note 3: Area of Occupancy (AOO) and Extent of Occurrence (EOO) method comparison

A comparison of the results based on the two range-size estimates [9] (AOO, EOO) reveals a highly correlated pattern of mean range-size and their relationship to plant taxa richness (Supplementary Figure 1; Supplementary Figure 2; Supplementary Figure 3). This indicates that the estimates of mean range-size are not influenced by these different calculation methods. Therefore, the simpler AOO method is presented in the main text results, and EOO results are presented here.

Supplementary Note 4: Time-windows and choice of grid cell size

We tested plant taxa richness to range-size relationships across various time-windows, ranging from 10,000-year (10ka) to 2,000-years (2ka), revealing a consistent pattern (Supplementary Figure 4). To incorporate more time-slices, thereby reducing noise from limited data, and to examine the relationship for the glacial and Holocene periods separately, 5,000-year (5ka) time-window is used in the main text.

During the calculation of the modern plant richness to range-size relationship across spatial scales, we tested this relationship (Supplementary Figure 5a, c, e, g) and compared taxon

range-size (Supplementary Figure 5b, d, f, h) using four grid cell sizes, ranging from 100 km × 100 km to 500 km × 500 km. The results consistently exhibited the same pattern. To mitigate the influence of any limited data resolution in GBIF, we use 200 km × 200 km grid cells in the main text.

Supplementary Note 5: Assessing the impact of extinction on the richness-range size relationship

To eliminate the effect of extinction, we used taxa with 100% assignment to the database in the main text analysis. Additionally, we tested the richness-range size relationship using taxa present in both the glacial and Holocene periods across northeast Siberia and Alaska, avoiding the impact of extinction during the glacial-interglacial transition. The results show the same pattern as in the main text (Supplementary Figure 7), confirming that extinction is not the main driver at the millennial time scale.

All data analyses in the main text and supplementary texts were done in R version 4.3.2 [10], with packages vegan [11], rgbif [12,13] , stats [10], arm [14], betapart [15], rioja [16], Hmisc [17], igraph package [18,19], dplyr [20], data.table [21], tidyr [22], sf [23,24], ggplot2 [25], viridis [26], cowplot [27], ggpubr [28], crayon [29], palaeoSig [30], and tidyverse [31].

Supplementary Figures

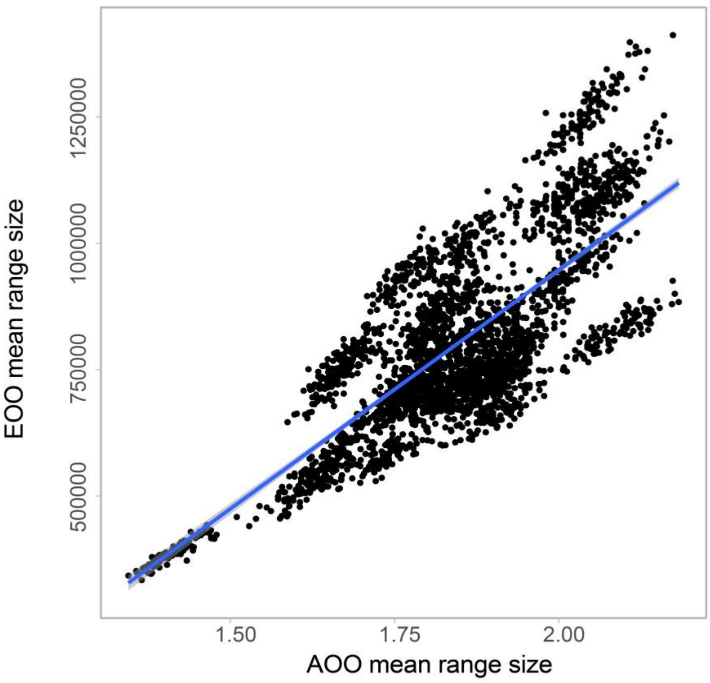

Supplementary Figure 1: Comparison of Area of Occupancy (AOO) and Extent of Occurrence (EOO) derived mean range-size ($t = 65.43$, $p<2\times10^{-16}$, $r^2 = 0.588$, $n=3000$). Source data are provided as a Source Data file.

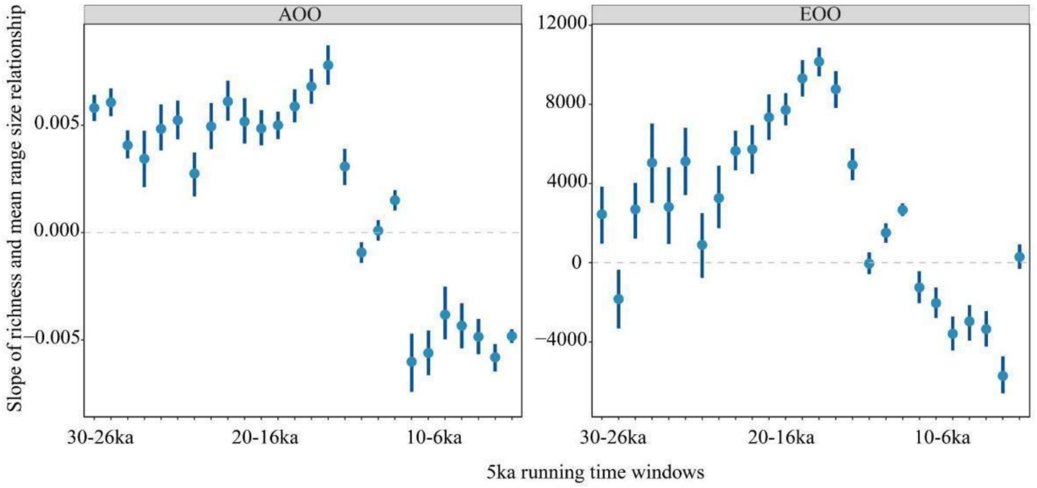

Supplementary Figure 2: Comparison of the slope of the richness to mean range-size relationship in 5,000-year time-windows based on the Area of Occupancy (AOO) and Extent of Occurrence (EOO) methods. Sample sizes are n=500 for most time-windows, except for “23–19ka”, “22–18ka”, “21–17ka”, “20–16ka”, and “19–15ka”, which have n=474. The middle point in the box corresponds to the mean value, and the whiskers show the minimum and maximum values for each time-window. Source data are provided as a Source Data file.

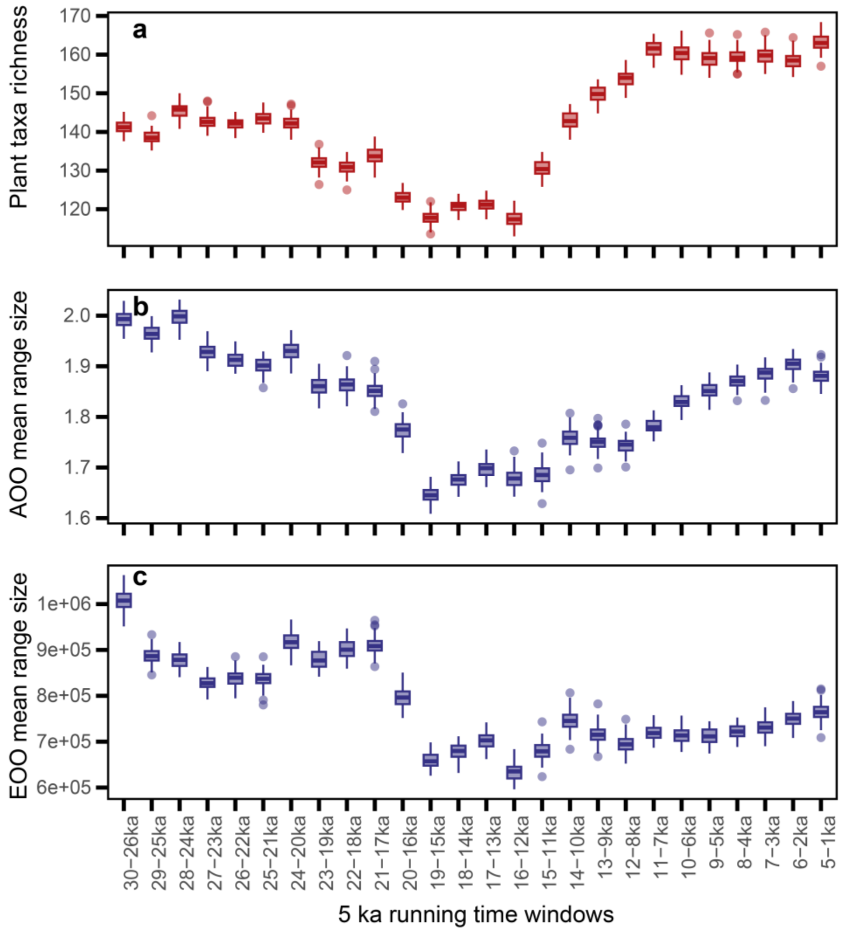

Supplementary Figure 3: Richness (a) and range-size change over 5,000-year running time-windows for the Area of Occupancy (AOO; b) and Extent of Occurrence (EOO) methods (c). Sample sizes are n=100 for all metrics. The boxplot is based on the result of 100 resampling iterations, where the middle line in the box corresponds to the median value, the edges of the box correspond to 25th (lower edge) and 75th (upper edge) percentile, and the ends of the whiskers correspond to the minimum and maximum values for each time-window. Source data are provided as a Source Data file.

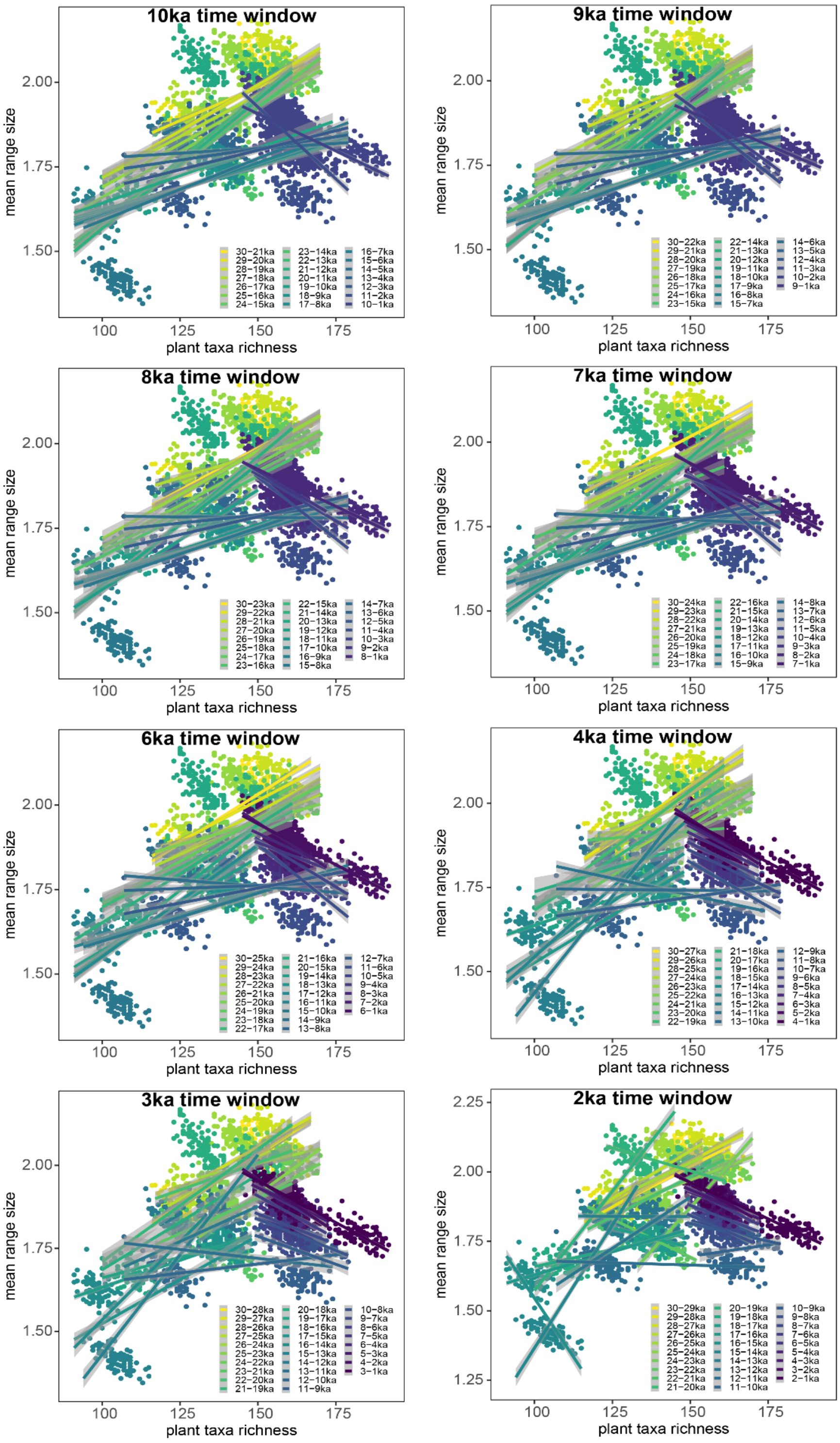

Supplementary Figure 4: Plant taxa richness to range-size relationships (with 95% confidence intervals) for time-windows ranging from 10,000-year (10 ka) to 2,000-year (2 ka), over the last 30,000 years, inferred from lake sedimentary ancient DNA collected from northeast Siberia and Alaska region. Coloured points show the richness and mean range-size of the 1,000-year time-slice samples (100 resampling iterations). The mean range-size is determined by calculating the average number of lakes occupied. Source data are provided as a Source Data file.

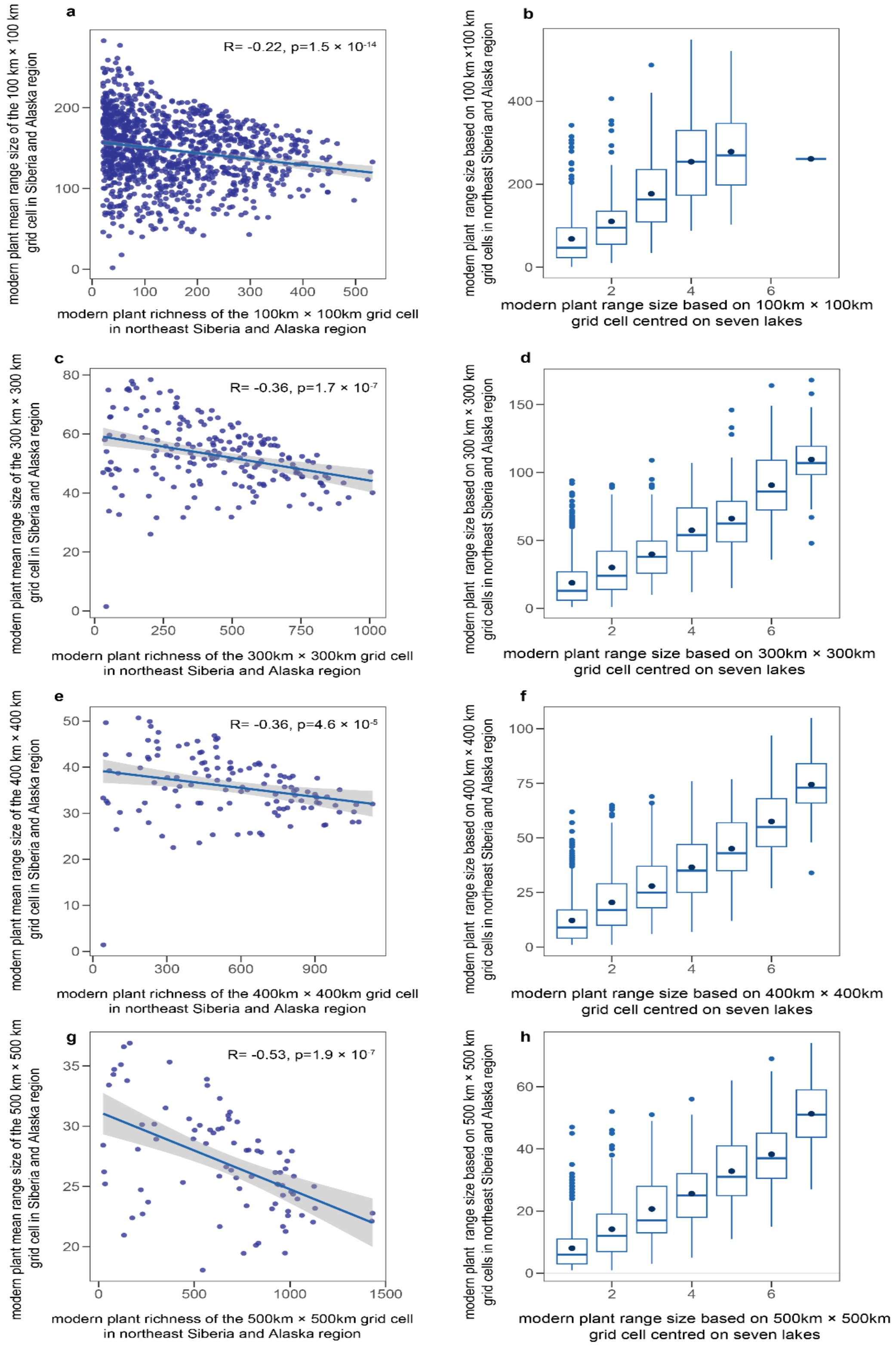

Supplementary Figure 5: The modern plant richness to range-size relationship across space in the northeast Siberia and Alaska region, based on modern plant taxa occurrences in grid cells of four sizes from 100 km × 100 km grid cells to 500 km × 500 km grid cells (a, c, e, g). Comparison of the same taxa range-size between two calculation methods: based on the sum of occupied grid cells centred on the seven

lakes, and the sum of occupied grid cells in northeast Siberia and Alaska region, for four grid cell sizes ranging from 100 km × 100 km to 500 km × 500 km (b, d, f, h). In the left panels, for each taxon, the range-size is defined as the sum of grid cells in which it occurred, for one grid cell, richness was determined by the number of taxa types, and the mean range-size was the average of all taxa ranges. Each point represents the plant richness and range-size for each grid cell. A linear regression is fitted to the points with a 95% confidence interval, and a Spearman's rank correlation coefficient was calculated to assess the relationship between plant richness and range size. In the right panels, the total taxa number is 1249, the middle line in the box corresponds to the median value, the middle point in the box corresponds to the mean value, the edges of the box correspond to 25th (lower edge) and 75th (upper edge) percentile, and the ends of the whiskers correspond to the minimum and maximum range-size based on the gird cells centred on the seven lakes. Source data are provided as a Source Data file.

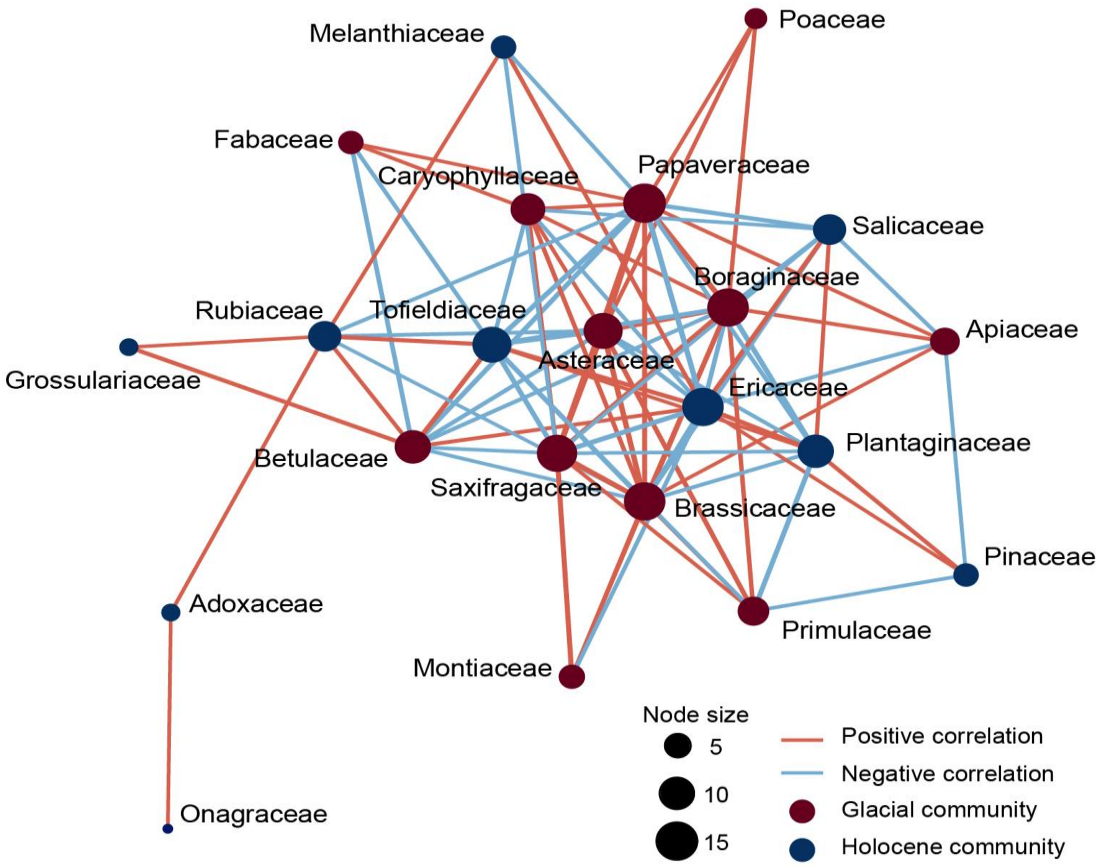

Supplementary Figure 6: Correlation network of plant groups. Plant families are represented by coloured nodes, where the size of the node represents the number of links (node degree), red nodes represent taxa belonging to the glacial community, and blue nodes represent the taxa belonging to the Holocene community. Blue edges indicate negative correlations between taxa, and red edges indicate positive correlations between taxa. Source data are provided as a Source Data file.

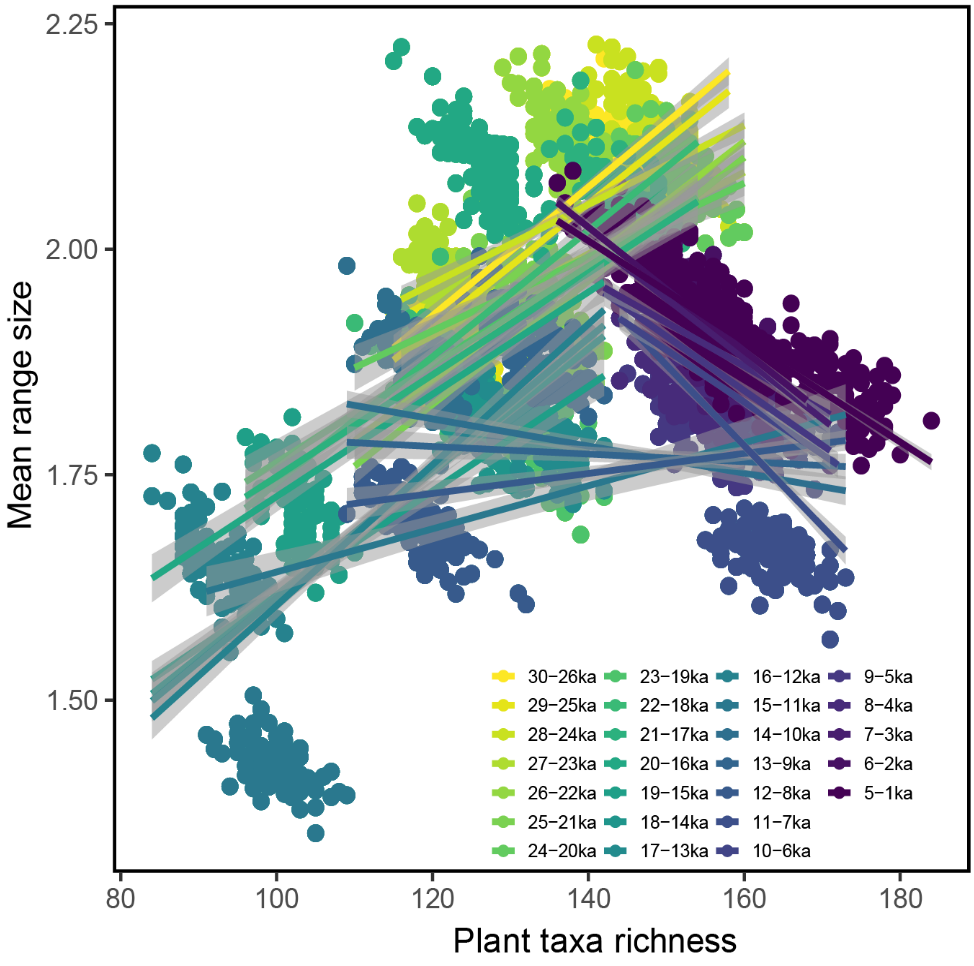

Supplementary Figure 7: Plant taxa richness to range-size relationships (with confidence intervals) per 5,000-year time-windows over the last 30,000 years, inferred from the taxa that occur in both the glacial and Holocene periods across northeast Siberia and Alaska. Coloured points show the richness and mean range-size of the 1,000-year time-slice samples (100 resampling iterations). The mean range-size is determined by calculating the average number of lakes occupied. Source data are provided as a Source Data file.

Supplementary Tables

Supplementary Table 1. The slope, adjusted R squared, and p-value of the richness to mean range-size (Area of Occupancy method) linear regression model based on 5,000-year running time-windows.

Time-window	Slope	R^2	P	Time-window	Slope	R^2	P
30-26ka	0.0058	0.5713	1.19×10^{-93}	17-13ka	0.0068	0.5084	8.22×10^{-79}
29-25ka	0.0061	0.5106	2.60×10^{-79}	16-12ka	0.0078	0.5027	1.44×10^{-77}
28-24ka	0.0041	0.3170	3.79×10^{-43}	15-11ka	0.0031	0.1512	1.71×10^{-19}
27-23ka	0.0034	0.0939	2.46×10^{-12}	14-10ka	-0.0009	0.0463	1.20×10^{-6}
26-22ka	0.0049	0.2023	2.89×10^{-26}	13-9ka	0.0001	0.0004	0.64
25-21ka	0.0052	0.3225	4.98×10^{-44}	12-8ka	0.0015	0.1252	3.46×10^{-16}
24-20ka	0.0028	0.0779	2.15×10^{-10}	11-7ka	-0.0060	0.2179	2.01×10^{-28}
23-19ka	0.0046	0.1895	2.46×10^{-23}	10-6ka	-0.0056	0.2968	$5.49 \times 10\text{-}^{40}$
22-18ka	0.0059	0.3008	1.40×10^{-38}	9-5ka	-0.0038	0.0952	1.73×10^{-12}
21-17ka	0.0050	0.1929	8.92×10^{-24}	8-4ka	-0.0043	0.1946	3.19×10^{-25}
20-16ka	0.0048	0.2702	3.60×10^{-34}	7-3ka	-0.0049	0.3553	2.01×10^{-49}
19-15ka	0.0052	0.4669	1.82×10^{-66}	6-2ka	-0.0058	0.5272	4.94×10^{-83}
18-14ka	0.0059	0.4390	1.64×10^{-64}	5-1ka	-0.0048	0.7631	7.23×10^{-158}

Supplementary Table 2. The slope, adjusted R squared, and p-value of the richness to mean range- size (Extent of Occurrence method) linear regression model based on 5,000-year running time-windows.

Time-window	Slope	R^2	P	Time-window	Slope	R^2	P
30-26ka	2433.3	0.0344	2.96×10^{-5}	17-13ka	10154.2	0.6978	1.70×10^{-131}
29-25ka	-1874.2	0.0200	1.63×10^{-3}	16-12ka	8779.8	0.5283	2.66×10^{-83}
28-24ka	2695.9	0.0457	1.42×10^{-6}	15-11ka	4939.4	0.3268	1.02×10^{-44}
27-23ka	5029.9	0.0773	2.49×10^{-10}	14-10ka	- 42.71	0.0001	0.85
26-22ka	2885.8	0.0300	1.03×10^{-4}	13-9ka	1507.2	0.1108	0.64
25-21ka	5128.8	0.1088	3.64×10^{-14}	12-8ka	2657.5	0.4771	4.02×10^{-72}
24-20ka	880.1	0.0037	0.174	11-7ka	-1231.9	0.0283	1.57×10^{-4}
23-19ka	2965.8	0.0435	4.68×10^{-6}	10-6ka	-2038.2	0.0987	6.47×10^{-13}
22-18ka	5628.8	0.2072	1.29×10^{-25}	9-5ka	-3588.8	0.1847	6.88×10^{-24}
21-17ka	5767.8	0.2116	3.36×10^{-26}	8-4ka	-2981.6	0.1158	5.16×10^{-15}
20-16ka	7596.0	0.4072	1.48×10^{-55}	7-3ka	-3343.9	0.1549	5.72×10^{-20}
19-15ka	8047.7	0.5417	5.45×10^{-82}	6-2ka	-5702.1	0.3261	1.31×10^{-44}
18-14ka	9291.3	0.5968	2.72×10^{-100}	5-1ka	276.5	0.0030	0.23

References

1. Müller, S. *et al.* Late Quaternary vegetation and environments in the Verkhoyansk Mountains region (NE Asia) reconstructed from a 50-kyr fossil pollen record from Lake Billyakh. *Quaternary Science Reviews* **29**, 2071–2086 (2010).
2. Courtin, J. *et al.* Vegetation Changes in Southeastern Siberia During the Late Pleistocene and the Holocene. *Frontiers in Ecology and Evolution* **9**, 625096 (2021).
3. Vachula, R. S. *et al.* Evidence of Ice Age humans in eastern Beringia suggests early migration to North America. *Quaternary Science Reviews* **205**, 35–44 (2019).
4. Baumer, M. M. *et al.* Climatic and environmental changes in the Yana Highlands of north-eastern Siberia over the last *c* . 57 000 years, derived from a sediment core from Lake Emanda. *Boreas* **50**, 114–133 (2021).
5. Vyse, S. A. *et al.* Geochemical and sedimentological responses of arctic glacial Lake Ilirney, Chukotka (far east Russia) to palaeoenvironmental change since ~51.8 ka BP. *Quaternary Science Reviews* **247**, 106607 (2020).
6. Vyse, S. A. *et al.* Sediment and carbon accumulation in a glacial lake in Chukotka (Arctic Siberia) during the Late Pleistocene and Holocene: combining hydroacoustic profiling and down-core analyses. *Biogeosciences* **18**, 4791–4816 (2021).
7. Lenz, M. *et al.* Climate and environmental history at Lake Levinson-Lessing, Taymyr Peninsula, during the last 62 kyr. *Journal of Quaternary Science* **37**, 836–850 (2022).
8. Taberlet, P. *et al.* Power and limitations of the chloroplast *trn*L (UAA) intron for plant DNA barcoding. *Nucleic Acids Research* **35**, e14–e14 (2007).
9. International Union for Conservation of Nature. IUCN Red List Categories and Criteria: Version 3.1. Second edition. Gland, Switzerland and Cambridge, UK: IUCN. iv + 32pp. (2012).
10. R Core Team. R: A Language and Environment for Statistical Computing. (2023).
11. Oksanen, J. *et al.* vegan: Community Ecology Package. (2022).
12. Chamberlain, S. *et al.* rgbif: Interface to the Global Biodiversity Information Facility API.

(2024).

13. Chamberlain, S. A. & Boettiger, C. R Python, and Ruby clients for GBIF species occurrence data. Preprint at https://doi.org/10.7287/peerj.preprints.3304v1 (2017).

14. Gelman, A. & Su, Y.-S. arm: Data Analysis Using Regression and Multilevel/Hierarchical Models. (2022).

15. Baselga, A. *et al.* betapart: Partitioning Beta Diversity into Turnover and Nestedness. (2023).

16. Steve Juggins. rioja: Analysis of Quaternary Science Data. (2023).

17. Harrell Jr, F. E. Hmisc: Harrell Miscellaneous. (2023).

18. Csárdi, G. *et al.* igraph for R: R interface of the igraph library for graph theory and network analysis. https://doi.org/10.5281/ZENODO.7682609 (2024).

19. Csárdi, G. *et al.* {igraph}: Network Analysis and Visualization in R.

20. Wickham, H., François, R., Henry, L., Müller, K. & Vaughan, D. dplyr: A Grammar of Data Manipulation. (2023).

21. Dowle, M. & Srinivasan, A. data.table: Extension of `data.frame`. (2023).

22. Wickham, H., Vaughan, D. & Girlich, M. tidyr: Tidy Messy Data. (2023).

23. Pebesma, E. & Bivand, R. *Spatial Data Science: With Applications in R*. (Chapman and Hall/CRC, New York, 2023). doi:10.1201/9780429459016.

24. Pebesma, E. Simple Features for R: Standardized Support for Spatial Vector Data. *The R Journal* **10**, 439 (2018).

25. Wickham, H. ggplot2: Elegant Graphics for Data Analysis. (2016).

26. Garnier, S. *et al.* viridis(Lite)-Colorblind-Friendly Color Maps for R. (2023).

27. Wilke, C. O. cowplot: Streamlined Plot Theme and Plot Annotations for ‘ggplot2’. (2020).

28. Kassambara, A. ggpubr: ‘ggplot2’ Based Publication Ready Plots. (2023).

29. Csárdi, G. crayon: Colored Terminal Output. (2022).

30. Telford, R. J. & Trachsel, M. palaeoSig: Significance Tests of Quantitative Palaeoenvironmental Reconstructions. (2023).

31. Wickham, H. *et al.* Welcome to the Tidyverse. *Journal of Open Source Software* **4**, 1686

(2019).
