# Supplementary material for: Plant interactions associated with a directional shift in the richness range size relationship during the Glacial-Holocene transition in the Arctic
Source: Nat Commun. 2025 Jan 28;16:1128. doi: 10.1038/s41467-025-56176-3 (PMC11775137; doi:10.1038/s41467-025-56176-3)
Supplement: Supplementary file 2 — Description of Additional Supplementary Files [file 41467_2025_56176_MOESM2_ESM.pdf]

## Description of Additional Supplementary Files

**File Name:** Supplementary Data 1

**Description:** Details of lakes and samples information
