# Supplementary material for: Plant interactions associated with a directional shift in the richness range size relationship during the Glacial-Holocene transition in the Arctic
Source: Nat Commun. 2025 Jan 28;16:1128. doi: 10.1038/s41467-025-56176-3 (PMC11775137; doi:10.1038/s41467-025-56176-3)
Supplement: Supplementary file 4 — Reporting Summary [file 41467_2025_56176_MOESM4_ESM.pdf]

nature portfolio

Corresponding author(s): Ulrike Herzschuh

Last updated by author(s): Sep 4, 2024

Reporting Summary

Nature Portfolio wishes to improve the reproducibility of the work that we publish. This form provides structure for consistency and transparency in reporting. For further information on Nature Portfolio policies, see our Editorial Policies and the Editorial Policy Checklist.

Statistics

For all statistical analyses, confirm that the following items are present in the figure legend, table legend, main text, or Methods section.

n/a	Confirmed	
☐	☒	The exact sample size (n) for each experimental group/condition, given as a discrete number and unit of measurement
☐	☒	A statement on whether measurements were taken from distinct samples or whether the same sample was measured repeatedly
☐	☒	The statistical test(s) used AND whether they are one- or two-sided<br>*Only common tests should be described solely by name; describe more complex techniques in the Methods section.*
☐	☒	A description of all covariates tested
☐	☒	A description of any assumptions or corrections, such as tests of normality and adjustment for multiple comparisons
☐	☒	A full description of the statistical parameters including central tendency (e.g. means) or other basic estimates (e.g. regression coefficient) AND variation (e.g. standard deviation) or associated estimates of uncertainty (e.g. confidence intervals)
☐	☒	For null hypothesis testing, the test statistic (e.g. F, t, r) with confidence intervals, effect sizes, degrees of freedom and P value noted<br>*Give P values as exact values whenever suitable.*
☒	☐	For Bayesian analysis, information on the choice of priors and Markov chain Monte Carlo settings
☒	☐	For hierarchical and complex designs, identification of the appropriate level for tests and full reporting of outcomes
☐	☒	Estimates of effect sizes (e.g. Cohen's d, Pearson's r), indicating how they were calculated

Our web collection on statistics for biologists contains articles on many of the points above.

Software and code

Policy information about availability of computer code

Data collection	The modern plant distribution data covered the study region and were downloaded from GBIF (https://www.gbif.org) using rgbif (version 3.7.8) to calculate the modern plant richness—mean range-size relationship.<br><br>To build the SibAla_2023 database, plant distribution information was also downloaded from GBIF (55-90°N, 50-150°E and 40-90°N, 150°E-140°W).<br><br>For all seven sediment cores, collection information was described in detail in the following papers:<br>Bilyakh PG1755: https://doi.org/10.1016/j.quascirev.2010.04.024<br>Bolshoe Toko PG2133: https://doi.org/10.3389/fevo.2021.625096<br>E5 E5-1A: https://doi.org/10.1016/j.quascirev.2018.12.003<br>Emanda C01412: https://doi.org/10.1111/bor.12476<br>Ilirney EN18208: https://doi.org/10.1016/j.quascirev.2020.106607<br>Ilirney 16-KP-01-L02: https://doi.org/10.1111/bor.12521<br>Levinson Lessing Co1401: https://doi.org/10.1002/jqs.3384<br>Rauchuagytgyn EN18218: https://doi.org/10.5194/bg-18-4791-2021<br><br>The pollen data used to reconstruct mean annual temperature was downloaded from the paper: https://doi.org/10.5194/essd-14-3213-2022.
Data analysis	R scripts for processing the data, with dataset input provided with this paper, can be downloaded via Zenodo: https://doi.org/10.5281/zenodo.11259177

DNA sequences were processed and assigned with the OBITools software (version 3.0.0).
All statistical analyses were conducted in R (version 4.3.2):
Sedimentary ancient DNA data resampling: R packages dplyr (version 1.1.3) and data.table (version 1.14.8);
Total plant richness, mean range size: R packages tidyr (version 1.3.0), dplyr (version 1.1.3), sf (version 1.0-14);
Environmental heterogeneity for each timeslice: R package betapart (version 1.6);
The relationship slope between richness and mean range size: R package arm (version 1.13-1);
Cushion and tree plant abundance: R package dplyr (version 1.1.3);
Binomial regression: R package stats (version 4.3.2);
Network analysis and display: R packages Hmisc (version 5.1-1), igraph (version 1.6.0), and ggraph (version 2.1.0);
Temperature reconstruction from pollen data: R packages rioja (version 1.0-6), crayon (version 1.5.2), palaeoSig (version 2.1-3), tidyverse (version 2.0.0).

For manuscripts utilizing custom algorithms or software that are central to the research but not yet described in published literature, software must be made available to editors and reviewers. We strongly encourage code deposition in a community repository (e.g. GitHub). See the Nature Portfolio guidelines for submitting code & software for further information.

Data

Policy information about availability of data

All manuscripts must include a data availability statement. This statement should provide the following information, where applicable:

- Accession codes, unique identifiers, or web links for publicly available datasets
- A description of any restrictions on data availability
- For clinical datasets or third party data, please ensure that the statement adheres to our policy

The raw sedaDNA sequence data have been deposited in the European Nucleotide Archive (ENA) at EMBL-EBI under accession number PRJEB76237 (https://www.ebi.ac.uk/ena/browser/view/PRJEB76237). The data generated in this study and used for analyses are provided in the Source Data file.

Research involving human participants, their data, or biological material

Policy information about studies with human participants or human data. See also policy information about sex, gender (identity/presentation), and sexual orientation and race, ethnicity and racism.

Reporting on sex and gender

Use the terms sex (biological attribute) and gender (shaped by social and cultural circumstances) carefully in order to avoid confusing both terms. Indicate if findings apply to only one sex or gender; describe whether sex and gender were considered in study design; whether sex and/or gender was determined based on self-reporting or assigned and methods used.
Provide in the source data disaggregated sex and gender data, where this information has been collected, and if consent has been obtained for sharing of individual-level data; provide overall numbers in this Reporting Summary. Please state if this information has not been collected.
Report sex- and gender-based analyses where performed, justify reasons for lack of sex- and gender-based analysis.

Reporting on race, ethnicity, or other socially relevant groupings

Please specify the socially constructed or socially relevant categorization variable(s) used in your manuscript and explain why they were used. Please note that such variables should not be used as proxies for other socially constructed/relevant variables (for example, race or ethnicity should not be used as a proxy for socioeconomic status).
Provide clear definitions of the relevant terms used, how they were provided (by the participants/respondents, the researchers, or third parties), and the method(s) used to classify people into the different categories (e.g. self-report, census or administrative data, social media data, etc.)
Please provide details about how you controlled for confounding variables in your analyses.

Population characteristics

Describe the covariate-relevant population characteristics of the human research participants (e.g. age, genotypic information, past and current diagnosis and treatment categories). If you filled out the behavioural & social sciences study design questions and have nothing to add here, write "See above."

Recruitment

Describe how participants were recruited. Outline any potential self-selection bias or other biases that may be present and how these are likely to impact results.

Ethics oversight

Identify the organization(s) that approved the study protocol.

Note that full information on the approval of the study protocol must also be provided in the manuscript.

Field-specific reporting

Please select the one below that is the best fit for your research. If you are not sure, read the appropriate sections before making your selection.

☐ Life sciences ☐ Behavioural & social sciences ☒ Ecological, evolutionary & environmental sciences

For a reference copy of the document with all sections, see nature.com/documents/nr-reporting-summary-flat.pdf

Ecological, evolutionary & environmental sciences study design

All studies must disclose on these points even when the disclosure is negative.

Study description	In this study, we attempt for the first time to detect the relationship between plant richness and mean range size in a temporal view within the constrained northeast Siberia and Alaska region, which avoids the inherent temporal dimensions restricted in spatial domain. The total plant richness, biotic environmental heterogeneity (beta-diversity), and mean range size were reconstructed for the last 30,000 years based on the sedimentary ancient DNA data from seven lakes. In order to understand the potential driver of the richness-mean range-size relationship and the mechanism, temperature was also reconstructed based on the pollen data in the study region, and network analysis was conducted to reconstruct plant interactions. Positive richness–mean range-size relationships were detected for the last glacial period, while the relationship shifts towards negative during the interglacial period. Based on the results, we conclude that neither speciation nor environmental heterogeneity acts as principal drivers. Network analyses show more positive plant interactions during the harsh glacial environment, exemplified by the facilitative effect of cushion plants, contributing to positive richness-mean range-size relationships. Conversely, during the warmer interglacial environment, more negative interactions occur with tree and shrub colonization, resulting in negative relationships.
Research sample	In total 352 sedaDNA samples from seven cores in the northeast Siberia and Alaska region were used in this study (Supplementary Table 1). For each sediment core, DNA extraction were conducted, each involving nine samples and one control (blank). In each PCR, both an extraction blank and a no template control (NTC) were incorporated to identify potential contamination during the extraction and PCR set-up. Additionally, for every extraction sample, three PCR replicates with uniquely tagged primers were performed. With the exception of the Bolshoe Toko core, replicates were amplified using the same tag combination. In each core, all extraction blanks and NTCs were included in the sequencing run, even though they yielded negative results in the PCRs.
Sampling strategy	For each of the lake sediment cores, the process of subsampling for sedaDNA analysis took place within the climate chamber at the Helmholtz Centre Potsdam-German Research Centre for Geosciences (GFZ), maintained at 4°C. This environment, devoid of any molecular genetic studies, was chosen to prevent potential contamination with modern DNA. Prior to working on the core, all surfaces in the climate chamber underwent thorough cleaning with DNA Exitus PlusTM and demineralized water. Additionally, sampling tools such as knives, scalpels, and their holders were meticulously cleaned before obtaining each sample, following the recommended procedures. To ensure sterility, approximately 3 mm of each sample slice that came into contact with the plastic tube or the thin foil covering the half-core was removed using a sterile scalpel.
Data collection	The modern plant distribution data covered the study region and were downloaded from GBIF (https://www.gbif.org) using rgbif (version 3.7.8) to calculate the modern plant richness–mean range-size relationship.<br><br>To build the SibAla_2023 database, plant distribution information was also downloaded from GBIF (55-90°N, 50-150°E and 40-90°N, 150°E-140°W).<br><br>For all seven sediment cores, collection information was described in detail in the following papers:<br>Bilyakh PG1755: https://doi.org/10.1016/j.quascirev.2010.04.024<br>Bolshoe Toko PG2133: https://doi.org/10.3389/fevo.2021.625096<br>E5 E5-1A: https://doi.org/10.1016/j.quascirev.2018.12.003<br>Emanda C01412: https://doi.org/10.1111/bor.12476<br>Ilirney EN18208: https://doi.org/10.1016/j.quascirev.2020.106607<br>Ilirney 16-KP-01-L02: https://doi.org/10.1111/bor.12521<br>Levinson Lessing Co1401: https://doi.org/10.1002/jqs.3384<br>Rauchuagytgyn EN18218: https://doi.org/10.5194/bg-18-4791-2021<br><br>The pollen data used to reconstruct mean annual temperature was downloaded from the paper: https://doi.org/10.5194/essd-14-3213-2022.
Timing and spatial scale	Our dataset cover the past 30,000 years and the northeast Siberia and Alaska region.
Data exclusions	For each sample, we performed quality checks on the composition signal of the three replicates' Amplicon Sequence Variants (ASVs). Replicates were excluded from the dataset if their total read count fell below 100. Furthermore, if a sample had fewer than three replicates or displayed different plant sequence types compared to the remaining replicates, those specific replicates were also excluded from the dataset.
Reproducibility	The archive lake sediment cores, along with DNA extraction samples and the prepared DNA amplicon-sequencing libraries, are stored at the Alfred Wegener Institute, Potsdam. The data analysis is reproducible, both the utilized data and scripts are available.
Randomization	Resample the read count randomly using the sample() function.
Blinding	The blinding test is not relevant to this study. We focus on plant richness and mean range-size relationship using sedimentary ancient DNA extracted from the lake core.

Did the study involve field work? ☒ Yes ☐ No

Field work, collection and transport

Field conditions	Description of field conditions while sediment core sampling are described in the individual publications:<br>Bilyakh PG1755: https://doi.org/10.1016/j.quascirev.2010.04.024<br>Bolshoe Toko PG2133: https://doi.org/10.3389/fevo.2021.625096<br>E5 E5-1A: https://doi.org/10.1016/j.quascirev.2018.12.003<br>Emanda C01412: https://doi.org/10.1111/bor.12476<br>Ilirney EN18208: https://doi.org/10.1016/j.quascirev.2020.106607<br>Ilirney 16-KP-01-L02: https://doi.org/10.1111/bor.12521<br>Levinson Lessing Co1401: https://doi.org/10.1002/jqs.3384<br>Rauchuagytgyn EN18218: https://doi.org/10.5194/bg-18-4791-2021
Location	The locations are described in the individual publications:<br>Bilyakh PG1755: https://doi.org/10.1016/j.quascirev.2010.04.024<br>Bolshoe Toko PG2133: https://doi.org/10.3389/fevo.2021.625096<br>E5 E5-1A: https://doi.org/10.1016/j.quascirev.2018.12.003<br>Emanda C01412: https://doi.org/10.1111/bor.12476<br>Ilirney EN18208: https://doi.org/10.1016/j.quascirev.2020.106607<br>Ilirney 16-KP-01-L02: https://doi.org/10.1111/bor.12521<br>Levinson Lessing Co1401: https://doi.org/10.1002/jqs.3384<br>Rauchuagytgyn EN18218: https://doi.org/10.5194/bg-18-4791-2021
Access & import/export	The related research grants and permits are acknowledged in the individual publications publications:<br>Bilyakh PG1755: https://doi.org/10.1016/j.quascirev.2010.04.024<br>Bolshoe Toko PG2133: https://doi.org/10.3389/fevo.2021.625096<br>E5 E5-1A: https://doi.org/10.1016/j.quascirev.2018.12.003<br>Emanda C01412: https://doi.org/10.1111/bor.12476<br>Ilirney EN18208: https://doi.org/10.1016/j.quascirev.2020.106607<br>Ilirney 16-KP-01-L02: https://doi.org/10.1111/bor.12521<br>Levinson Lessing Co1401: https://doi.org/10.1002/jqs.3384<br>Rauchuagytgyn EN18218: https://doi.org/10.5194/bg-18-4791-2021
Disturbance	n/a

Reporting for specific materials, systems and methods

We require information from authors about some types of materials, experimental systems and methods used in many studies. Here, indicate whether each material, system or method listed is relevant to your study. If you are not sure if a list item applies to your research, read the appropriate section before selecting a response.

Materials & experimental systems

n/a	Involved in the study	
☒	☐	Antibodies
☒	☐	Eukaryotic cell lines
☒	☐	Palaeontology and archaeology
☒	☐	Animals and other organisms
☒	☐	Clinical data
☒	☐	Dual use research of concern
☒	☐	Plants

Methods

n/a	Involved in the study	
☒	☐	ChIP-seq
☒	☐	Flow cytometry
☒	☐	MRI-based neuroimaging

Plants

Seed stocks	*Report on the source of all seed stocks or other plant material used. If applicable, state the seed stock centre and catalogue number. If plant specimens were collected from the field, describe the collection location, date and sampling procedures.*
Novel plant genotypes	*Describe the methods by which all novel plant genotypes were produced. This includes those generated by transgenic approaches, gene editing, chemical/radiation-based mutagenesis and hybridization. For transgenic lines, describe the transformation method, the number of independent lines analyzed and the generation upon which experiments were performed. For gene-edited lines, describe the editor used, the endogenous sequence targeted for editing, the targeting guide RNA sequence (if applicable) and how the editor was applied.*
Authentication	*Describe any authentication procedures for each seed stock used or novel genotype generated. Describe any experiments used to assess the effect of a mutation and, where applicable, how potential secondary effects (e.g. second site T-DNA insertions, mosiacism, off-target gene editing) were examined.*
